# Functions of resolvin D1-ALX/FPR2 receptor interaction in the hemoglobin-induced microglial inflammatory response and neuronal injury

**DOI:** 10.1186/s12974-020-01918-x

**Published:** 2020-08-14

**Authors:** Guang-Jie Liu, Tao Tao, Han Wang, Yan Zhou, Xuan Gao, Yong-Yue Gao, Chun-Hua Hang, Wei Li

**Affiliations:** 1 Department of Neurosurgery, Nanjing Drum Tower Hospital, The Affiliated Hospital of Nanjing University Medical School, Nanjing, China; 2 Department of Neurosurgery, Nanjing Drum Tower Hospital Clinical College of Nanjing Medical University, Nanjing, China; 3 Department of Neurosurgery, Jinling Hospital, School of Medicine, Southern Medical University (Guangzhou), Nanjing, China

**Keywords:** Subarachnoid hemorrhage, Early brain injury, Resolvin D1, Lipoxin A4 receptor/Formyl peptide receptor 2, Microglial inflammation, Neuronal death

## Abstract

**Background:**

Early brain injury (EBI) has been thought to be a key factor affecting the prognosis of subarachnoid hemorrhage (SAH). Many pathologies are involved in EBI, with inflammation and neuronal death being crucial to this process. Resolvin D1 (RvD1) has shown superior anti-inflammatory properties by interacting with lipoxin A4 receptor/formyl peptide receptor 2 (ALX/FPR2) in various diseases. However, it remains not well described about its role in the central nervous system (CNS). Thus, the goal of the present study was to elucidate the potential functions of the RvD1-ALX/FPR2 interaction in the brain after SAH.

**Methods:**

We used an in vivo model of endovascular perforation and an in vitro model of hemoglobin (Hb) exposure as SAH models in the current study. RvD1 was used at a concentration of 25 nM in our experiments. Western blotting, quantitative polymerase chain reaction (qPCR), immunofluorescence, and other chemical-based assays were performed to assess the cellular localizations and time course fluctuations in ALX/FPR2 expression, evaluate the effects of RvD1 on Hb-induced primary microglial activation and neuronal damage, and confirm the role of ALX/FPR2 in the function of RvD1.

**Results:**

ALX/FPR2 was expressed on both microglia and neurons, but not astrocytes. RvD1 exerted a good inhibitory effect in the microglial pro-inflammatory response induced by Hb, possibly by regulating the IRAK1/TRAF6/NF-κB or MAPK signaling pathways. RvD1 could also potentially attenuate Hb-induced neuronal oxidative damage and apoptosis. Finally, the mRNA expression of IRAK1/TRAF6 in microglia and GPx1/bcl-xL in neurons was reversed by the ALX/FPR2-specific antagonist Trp-Arg-Trp-Trp-Trp-Trp-NH2 (WRW4), indicating that ALX/FPR2 could mediate the neuroprotective effects of RvD1.

**Conclusions:**

The results of the present study indicated that the RvD1-ALX/FPR2 interaction could potentially play dual roles in the CNS, as inhibiting Hb promoted microglial pro-inflammatory polarization and ameliorating Hb induced neuronal oxidant damage and death. These results shed light on a good therapeutic target (ALX/FPR2) and a potential effective drug (RvD1) for the treatment of SAH and other inflammation-associated brain diseases.

## Introduction

Subarachnoid hemorrhage (SAH) is a serious hemorrhagic disease of the central nervous system (CNS). Although SAH only accounts for 5% of stroke, it has attracted a great deal of attention owing to the high rate of disability and mortality associated with this disease [1, 2]. It is currently believed that early brain injury (EBI) is an important factor affecting the prognosis of SAH [3]. EBI is associated with many pathologies, such as blood–brain barrier interruption, brain edema, inflammation, and neuronal death [2]. Due to the complex mechanisms of EBI following SAH, no drugs are currently available to effectively treat EBI, leading to continued research to identify potential effective drugs and targets [4].

RvD1 (7S,8R,17S-trihydroxy-4Z,9E,11E,13Z,15E,19Z-docosahexaenoic acid) is a metabolite of docosahexaenoic acid (DHA) identified by Serhan [5] that appears in the resolution phase of the inflammatory response [6]. RvD1 has been shown to exert good regulatory effects on inflammation, evidenced by its ability to limit the recruitment or infiltration of neutrophils [7, 8], to inhibit the production of pro-inflammatory factors [9, 10], and to promote neutrophil apoptosis and their efferocytosis by macrophages [11]. These functions may be mediated by ALX/FPR2 [12], a type of G-protein-coupled receptors (GPCR). The FPR family comprises FPR, FPRL1, and FPRL2, which were first cloned from human DNA [13]. Orthologs of these human genes were identified in mice. FPR1 is the mouse ortholog of human FPR, and ALX/FPR2 is similar to human FPRL1, although no ortholog of FPRL2 has been identified in mice. In rats, the ortholog of ALX/FPR2 was cloned by Chiang et al. [14]. The FPR family is involved in immune response against pathogens or microbes. FPR1 mediates leukocyte chemotaxis, responding to formyl peptides derived from bacteria or mitochondria [13]. ALX/FPR2 is promiscuous and can bind peptides or lipids, such as the neuroprotective peptide humanin, the chemokine variant sCKβ8-1, and lipoxin A4. The ALX/FPR2 signaling pathway involves both pro-inflammatory and pro-resolution effects, depending on the ligand [15]. In addition, the interaction between RvD1 and ALX/FPR2 has been confirmed using a GPCR-β-arrestin-coupled system in human phagocytes by Krishnamoorthy and colleagues [16].

Microglia are the primary cells responsible for brain inflammation in EBI after SAH. Microglia can be activated by many substances, such as red cell lysis products, hemoglobin, and high mobility group protein 1 (HMGB1). Once activated, microglia can polarize to different phenotypes with different functions [17–19]. There are two primary types of polarization, including pro-inflammatory polarization, which involves the production of many pro-inflammatory factors (TNF-α, IL-1β, CD86, and iNOS), and anti-inflammatory polarization, which is characterized by the expression of many anti-inflammatory- or phagocytosis-related genes (IL-10, Arg1, and CD206). Several studies have shown that neurological function after SAH can be improved by regulating microglial polarization (through the promotion of anti-inflammatory polarization or the inhibition of pro-inflammatory polarization) [20–22]. However, few studies have investigated the specific effects of RvD1 on microglia. RvD1 has been shown to inhibit TNF-α and IL-1β secretion as well as NF-κB pathway activation in LPS-stimulated microglia in vitro [23], although LPS is a bacterial-derived substance, which is not consistent with the aseptic inflammatory response after SAH observed in this study. RvD1 has also been observed to enhance IL-4-induced anti-inflammatory polarization of the microglial cell line BV2, which was achieved by enhancing the nuclear transfer and DNA binding ability of PPARγ [24]. What is more, these effects could be blocked by ALX/FPR2 inhibitors. Therefore, we speculated that RvD1 might also regulate the microglial polarization induced by Hb.

Neuronal injury or death is the key determinant of poor prognosis after SAH with neurons demonstrated to undergo significant apoptosis in the cortex, subcortical, and hippocampal areas [25]. The factors that cause neuronal apoptosis are highly complex, which include cerebral ischemia [26, 27], microcirculation failure [28], subarachnoid blood stimulation [29], and inflammation [30]. For example, some studies have shown that the inflammatory factor TNF-α can significantly activate the TNF-α receptor of neurons and activate the downstream apoptosis pathway to induce neuronal death [30]. However, few studies have investigated whether ALX/FPR2 could function in neurons. ALX/FPR2 activation has been shown to promote the growth of neuronal axons and dendrites [31], and some studies have demonstrated that treatment with ALX/FPR2 agonists could promote neural stem cell migration and differentiation, which were achieved by promoting F-actin aggregation [32]. Therefore, the goal of the current study was to determine whether RvD1 can inhibit the apoptosis or synaptic damage of neurons after SAH.

## Materials and methods

### Reagents

RvD1 (CAS. no. 872993-05-0, Cayman Chemical Company, MI, USA), Hb (Sigma, Darmstadt, Germany), and WRW4 (cat. no. 2262, Tocris Bioscience, MO, USA) were purchased from the indicated commercial suppliers. The chemical structure of RvD1 is shown in the Supplementary Material Fig. S1. RvD1 was added 30 min before Hb stimulation according to the previous published articles [24, 33, 34]. Concentration of RvD1 used in the present study was based on the in vitro experiments in macrophages [16], BV2 microglial cell lines [24], and primary alveolar epithelial type 2 cells [35]. Meanwhile, we also did a simple dose response experiment in microglia and neurons, respectively (Supplementary Material Fig. S2). At last, the concentration of 25 nM was chosen for the whole experiment.

### Animal experiments

Twenty-eight male Sprague-Dawley rats (RRID: RGD_10395233) weighing 270–310 g were purchased from the Animal Core Facility of Nanjing Medical University. Our study was approved by the Experimental Animal Ethics Committee of Nanjing Drum Tower Hospital (approved number: 2018020003). Rats were maintained in a comfortable environment at a constant temperature of 26 ± 2 °C with a 12-h light/dark cycle and free access to water and a standard chow diet.

The endovascular perforation model of SAH in rats was generated as described in a previous study [36]. Briefly, rats were trans-orally intubated and mechanically ventilated with 3% isoflurane anesthesia during the operation. A 4–0 monofilament nylon suture was inserted from the external carotid artery into the right internal carotid artery, after which the bifurcation of the anterior and middle cerebral arteries was punctured. Similar procedures were performed for the sham operation group, but the suture was withdrawn without artery perforation. A heated blanket was used to warm the rats until they recovered from anesthesia.

Rats were killed by isoflurane anesthesia at each time point followed by decapitation and the removal of the brains. The basal cortex tissue was sampled and stored at – 80 °C.

### Primary microglial cell culture

Primary microglial cells from the cerebral cortex were cultured as previously described [37]. Briefly, the meninges of the brains from neonatal (1 day) mice were carefully removed and then digested in 0.25% trypsin (Gibco, USA) at 37 °C for 10 min. Subsequently, the tissue was triturated with warm culture medium and filtered through a 70-μm strainer (Sigma). After the suspension was centrifuged at 1000 r/min for 5 min, the remaining cells were resuspended in Dulbecco’s modified Eagle’s medium (DMEM, GIBCO, USA) supplemented with 10% fetal bovine serum (FBS, Biological Industries, USA). Then, the cells were seeded into flasks, and after approximately 10 days, when the glial cells reached confluency, the flasks were shaken and non-adherent cells were collected and transferred to plates to obtain microglia. Approximately 2 days after seeding onto plates, the microglia were in a resting state and could be used for experiments.

### Primary neuron culture

For neuron cultures, the cerebral cortex from a fetal rat at embryonic day 18 was used. The culture protocols were essentially the same as those describe above, except that the cells were seeded in plates pre-coated with 0.1 mg/ml poly-D-lysine. Four hours after seeding, the medium was completely replaced with neurobasal medium supplemented with 1% GlutMax (GIBCO, USA) and 2% B27. Subsequently, after 7 days of cultivation, neurons were available for experiments [38].

### Western blotting analyses

For brain tissues, proteins were extracted with lysis buffer (Thermo Fisher Scientific, USA) at a volume of 10 μl/mg of tissue. For cell proteins, the cells were washed twice with phosphate-buffered saline (PBS) and then mixed with 100 μl lysis buffer per well to extract the proteins. Equal amounts of protein were then run on 10% SDS polyacrylamide gels and subsequently transferred to polyvinylidene difluoride membranes (Sigma). After blocking the membranes with 5% skim milk for 1 h, they were incubated with specific antibodies at 4 °C overnight. Primary antibodies against the total or phosphorylated forms of the following proteins were purchased from Cell Signaling Technology (CST, USA): p65 (1:1000, cat. no. D14E12), P-p65 (1:1000, cat. no. 93H1), jnk (1:2000, cat. no. 4668S), P-jnk (cat. no. 81E11), p44/42 (1:1000, cat. no. 137F5), P-p44/42 (1:1000, cat. no. D13.14.4E), p38 (1:1000, cat. no. D13E1), P-38 (1:1000, cat. no. D3F9). In addition, antibodies against the following proteins were also used: ALX/FPR2 (1:1000, cat. no. ab203129, Abcam), bax (1:1000, D2E11, CST), bcl-xL (1:1000, 54H6, CST), cleaved caspase-3 (1:1000, 5A1E, CST), caspase-3 (1:1000, D3R6Y, CST), and β-actin (1:2000 cat. no. BS6007M, Bioworld Technology, Minneapolis, MN, USA). The membranes were then incubated with the corresponding horseradish peroxidase (HRP)-conjugated IgG (cat. no. BS13278 or BS30503, Bioworld Technology) for 1 h at room temperature after being washed three times with Tris-buffered saline with Tween (TBST). Protein signals were developed with a chemiluminescence solution (cat. no. 46640, Thermo Fisher Scientific), and ImageJ software (RRID: SCR_003070) was used for band intensity quantification.

### ELISA

Primary microglial cells in 24-well plates were pre-incubated with 25 nM RvD1 for 30 min, which was followed by the addition of Hb (20 μM) and another incubation for 1, 4, 12, and 24 h. Subsequently, the culture medium was centrifuged to obtain the supernatants which were then stored at – 80 °C. The protein levels of TNF-α and IL-10 were detected by ELISA (Multi Sciences Biotech, Hangzhou, China) according to the manufacturer’s instructions.

### Quantitative PCR (qPCR)

Primary microglial cells in 12-well plates were pre-incubated with 25 nM RvD1 for 30 min, which was followed by treatment with Hb (20μM) for 1, 4, 12, and 24 hours, respectively. Primary neurons were pre-incubated with 25 nM RvD1 for 30 min, which was followed by treatment with Hb (50 μM) for 12 h. Total RNA was extracted from cells using TRIzol reagent following the manufacturer’s instructions. After quantifying the concentration and purity of the extracted RNA with a BioPhotometer (Eppendorf, Germany), 1.0 μg of RNA was reverse transcribed to cDNA with a reverse transcription mix (Vazyme, Nanjing, China). Finally, qPCR was performed with SYBER Green mix (Roche, Switzerland) and a PCR thermocycler system (Applied Biosystems, USA). The primers used for qPCR are shown in Table 1. An internal control (GAPDH) was used to normalize the expression of each gene, and the 2^−ΔΔCt^ method [39, 40] was used to determine the relative gene expression.

**Table 1:** Primer pairs used in quantitative PCR

Primer name	Sequence	
Mice IL-1β	Forward	AAGCCTCGTGCTGTCGGACC
	Reverse	TGAGGCCCAAGGCCACAGG
Mice TNF-α	Forward	CAAGGGACAAGGCTGCCCCG
	Reverse	GCAGGGGCTCTTGACGGCAG
Mice iNOS	Forward	CAGCTGGGCTGTACAAACCTT
	Reverse	CATTGGAAGTGAAGCGTTTCG
Mice CD86	Forward	TCTCCACGGAAACAGCATCT
	Reverse	CTTACGGAAGCACCCATGAT
Mice IL-10	Forward	TAGAGCTGCGGACTGCCTTC
	Reverse	AGAAATCGATGACAGCGCCTC
Mice Arg1	Forward	CAGAAGAATGGAAGAGTCAG
	Reverse	CAGATATGCAGGGAGTCACC
Mice CD206	Forward	TCAGACGAAATCCCTGCTACTG
	Reverse	AGCCTGACCCCAACTTCTCG
Mice IRAK1	Forward	CCACCCTGGGTTATGTGCC
	Reverse	GAGGATGTGAACGAGGTCAGC
Mice TRAF6	Forward	AAAGCGAGAGATTCTTTCCCTG
	Reverse	ACTGGGGACAATTCACTAGAGC
Mice GAPDH	Forward	TCCCAGCTTAGGTTCATCAGGT
	Reverse	TACGGGACGAGGAAACACTCTC
Rat Ho-1	Forward	TGCTCGCATGAACACTCTG
	Reverse	TCCTCTGTCAGCAGTGCCT
Rat GPx1	Forward	CACAGTCCACCGTGTATGCC
	Reverse	AAGTTGGGCTCGAACCCACC
Rat Bcl-xL	Forward	AGGATACAGCTGGAGTCAG
	Reverse	TCTCCTTGTCTACGCTTTCC
Rat Bax	Forward	CTGCAGAGGATGATTGCTGA
	Reverse	GATCAGCTCGGGCACTTTAG
Rat GAPDH	Forward	CAAGTTCAACGGCACAGTCA
	Reverse	CCCCATTTGATGTTAGCGGG

### Immunofluorescence staining

Immunofluorescence staining was performed according to our previously published protocols [41, 42]. Briefly, the brain sections (6 μm thick) were incubated with primary antibodies against ALX/FPR2 (1:200, cat. no. ab203129, Abcam), GFAP (1:200, cat. no. MAB3402X, EMD Millipore, USA), Iba-1 (1:200, RRID: AB_2224402), and NeuN (1:200, cat. no. ABN78A4, EMD Millipore, USA) overnight at 4 °C. Then, the brain sections were incubated with the corresponding secondary antibodies conjugated with Alexa Fluor 488 and/or Alexa Fluor 594 (Jackson ImmunoResearch Incorporation, West Grove, PA, USA) for 1 h at room temperature, after which a ZEISS HB050 inverted microscope system was used for fluorescence detection.

For in vitro cell staining, primary antibodies against Iba-1 (1:200, RRID: AB_2224402) and p65 (1:200, 1:1000, cat. no. D14E12, CST) were used for microglia staining, while a primary antibody against MAP2 (1:500, cat. no. ab183830, Abcam) was used to stain neurons. The protocols and the secondary antibodies used for in vitro cell staining were the same as those used to stain brain sections.

### Cell viability assay

Approximately 2 × 10^^4^ primary neurons per well were seeded into 96-well plates, and after 7 days, the cells were treated with 25 nM RvD1 for 30 min followed by Hb stimulation for 24 h. Then, the medium was completely replaced with new medium containing 10% of the Cell Counting Kit-8 (CCK-8) reagent (Dojindo Laboratories, Kumamoto, Japan) and incubated for 2 h at 37 °C. Subsequently, the absorbance at 450 nm was measured, and the results were calculated as the relative cell viability.

### Live/dead cell double staining assay

A Live/Dead Cell Double Staining Kit (KGAF001, KeyGEN BioTECH, Jiangsu, China) was used to assess cell viability following the manufacturer’s instructions. First, 5 μl of reagent A (PI) and B (calcium AM) were mixed with 10 ml of PBS. Then, the cells were washed twice with sterile pre-heated PBS, the prepared staining solution described above was added, and the cells were incubated at room temperature for 5–10 min. Finally, the cells were washed twice with PBS and then observed under a fluorescence microscope in time.

### MDA content detection

The total tissue protein was extracted with PBS buffer, and the required reagents were added according to the manufacturer’s instructions (S0131, Beyotime, Shanghai, China). First, 100 μl of sample or standard sample was added to a centrifuge tube, followed by addition of 200 μl of MDA detection solution addition. After being heated at 100 °C for 15 min, the mixture was centrifuged at 12000×*g* at room temperature for 15 min, and then 200 μl of the resulting supernatant was transferred to a 96-well plate. The MDA concentration was read at a wavelength of 530–540 nm and was calculated according to the standard curve.

### SOD enzyme activity test

Total tissue protein was extracted in PBS buffer, and the required reagents were prepared according to the manufacturer’s instructions (S0101, Beyotime, Shanghai, China). Reagent 1 (1 ml), reagent 2 (0.1 ml), reagent 3 (0.1 ml), reagent 4 (0.1 ml), standard sample (0.5m l), and sample (0.5ml) were added to the centrifuge tube respectively and placed at room temperature for 10 min. Then, the absorbance in each well was measured at 550 nm, and the SOD activity in the sample was calculated according to the standard curve.

### ROS detection

The ROS content in primary neurons was determined using the DCFH-DA (D6883, Sigma) method. The original culture medium of neurons was replaced with DMEM medium supplemented with 10 μM DCFH-DA. Then, after incubating in an incubator for 20 min, the medium was removed, and the cells were washed three times with preheated DMEM without DAFH-DA. Then, the ROS content in cells was immediately observed under an inverted fluorescence microscope.

### Statistical analysis

GraphPad Prism (RRID: SCR_002798) Windows version 8.0 was used to perform statistical analyses. Two experimental groups were compared by two-tailed unpaired Student’s *t* tests. Three or more groups were compared by one-way ANOVA followed by post hoc Tukey’s tests. Two-way ANOVA was used to analyze the interaction effect of time courses and treatments. Differences were considered significant at *P* < 0.05, presented as * or #, ns: not significant. No outlier tests were performed in the present study, and no statistical methods were used to predetermine the sample size. The data are presented as the means ± SD.

## Results

### ALX/FPR2 is elevated after SAH and primarily expresses in neurons and microglia, rather than astrocytes

**Fig. 1:**
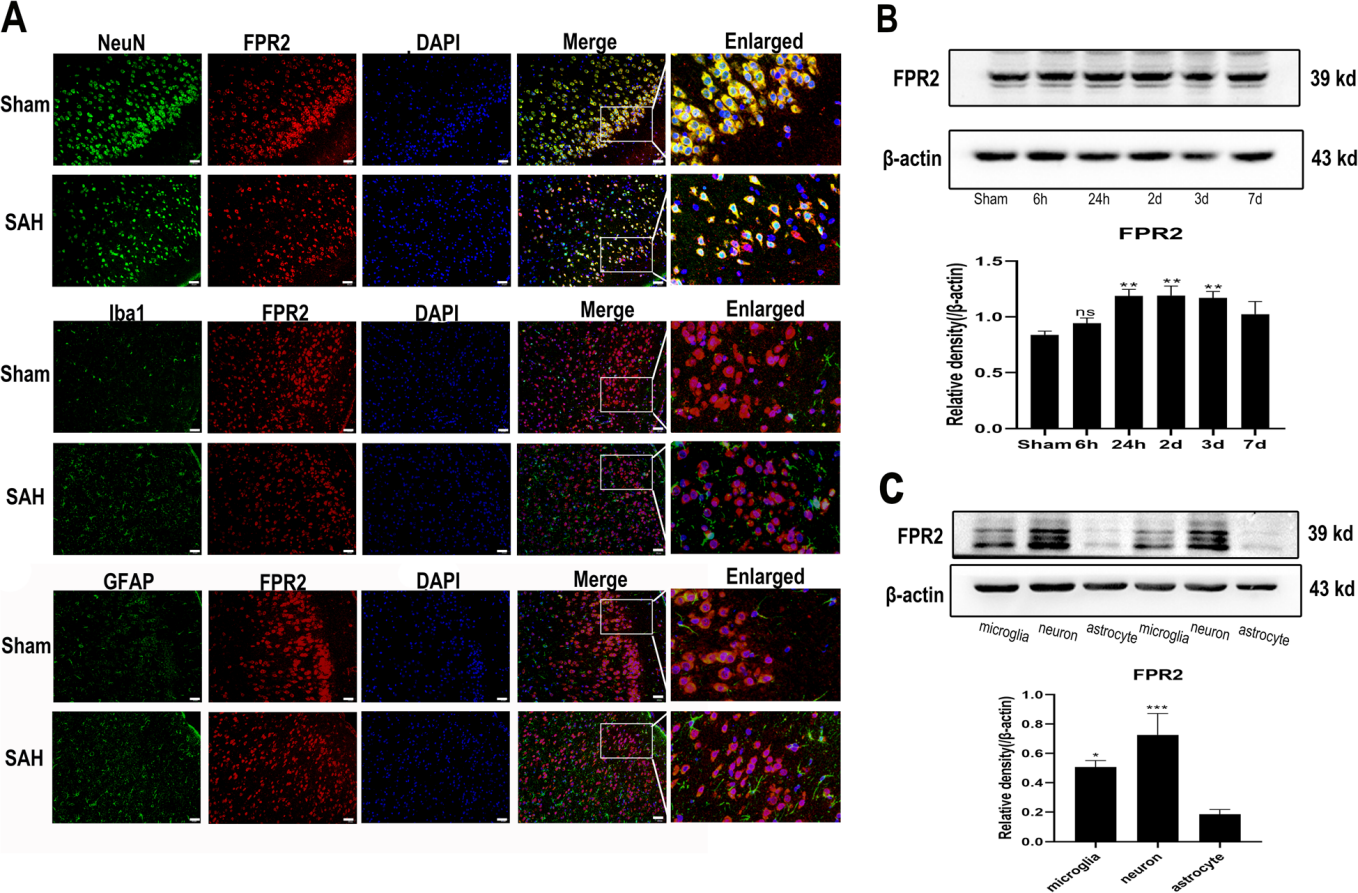
ALX/FPR2 expression location and time course change after SAH. **a** ALX/FPR2 was co-stained for NeuN (a neuronal marker), Iba1 (a microglial marker), and GFAP (an astrocytic marker) (*n* = 3). **b** Fluctuation of ALX/FPR2 expression at different time points after SAH (*n* = 3). **c** ALX/FPR2 expression pattern in primary microglia, neurons, and astrocytes (*n* = 5). The data were analyzed by one-way ANOVA and Tukey’s post hoc multiple comparison. *p < 0.05, ***p < 0.001 compared with the sham group for **b** or with the astrocyte group for **c.** Bar = 50μm. *n* is the number of animals or independent cell samples

The location of ALX/FPR2 expression has remained controversial. Therefore, we conducted double immunofluorescence and western blot analyses to observe which cells expressed ALX/FPR2. As shown in Fig. 1a, the rat cerebral cortex staining results showed that ALX/FPR2 was highly expressed in neurons (marked by NeuN), whereas little expression was observed in microglia (marked by Iba1) and astrocytes (marked by GFAP). However, different results were obtained from in vitro experiments (Fig. 1c), where ALX/FPR2 exhibited the highest expression in primary neuron, followed by primary microglia, while primary astrocytes did not exhibit ALX/FPR2 expression. Regarding the fluctuation in ALX/FPR2 expression after SAH over time, as shown in Fig. 1b, the expression of ALX/FPR2 protein in the rat brain significantly increased from 24 h to 3 days before decreasing on the 7th day after SAH.

### Hb induces significant microglial pro-inflammatory polarization

**Fig. 2:**
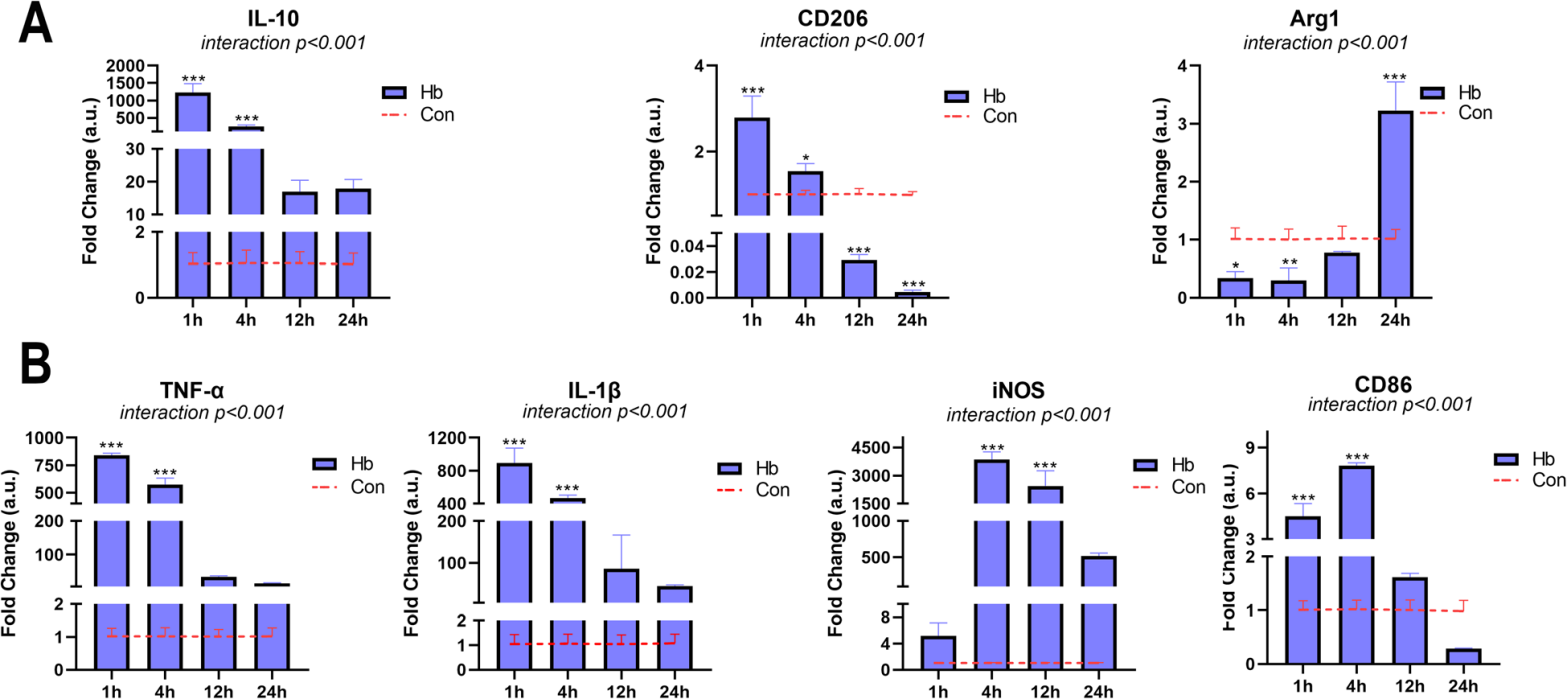
Effects of Hb on the polarization of primary microglia. Microglia were cultured in medium containing 20 μM Hb for 1–24 h. **a** The mRNA expression of anti-inflammatory markers (IL-10, CD206, and Arg1) (*n* = 3). **b** The mRNA expression of pro-inflammatory markers (TNF-α, IL-1β, iNOS, and CD86) (*n* = 3). The data are shown as the relative changes of the experimental group versus the control group (baseline) and were analyzed by two-way ANOVA followed by Sidak post hoc multiple comparison, *p < 0.05, **p < 0.01, and ***p < 0.001. *n* is the number of independent cell samples

We constructed the SAH model of primary microglia by Hb stimulation in vitro and performed qPCR to assess mRNA expression of related genes. As shown in Fig. 2, under the stimulation of 20 μM Hb, the primary microglia were obviously activated, as evidenced by obvious changes in the polarization phenotype index. For pro-inflammatory polarization (Fig. 2b), the trend of the changes in TNF-α and IL-1β cytokine levels was essentially the same. The transcription of these genes significantly increased and peaked from 1 h after Hb stimulation, and gradually decreased with levels remaining higher than that of the control group. iNOS expression was not significantly altered at 1 h but increased and peaked at 4 h before gradually decreasing. CD86 was labeled on the cell membrane surface, with levels significantly increasing at 1 hour and peaking at 4 hours before gradually decreasing, but the overall increase was not as obvious as that of the previous markers. For anti-inflammatory polarization (Fig. 2a), IL-10 showed a time course expression pattern similar to that of TNF-α and IL-1β, peaking after 1 h before gradually decreasing until the 24-h time point. CD206 expression also peaked after 1 h, exhibiting higher levels than that observed in the control group after 4 h, although its expression sharply decreased from 12 to 24 h. Arg1 expression gradually increased after a significant decrease after 1 h and rebounded at the 24-h time point, which was significantly higher than that of the control group.

### RvD1 attenuates Hb-induced microglial pro-inflammatory polarization

**Fig. 3:**
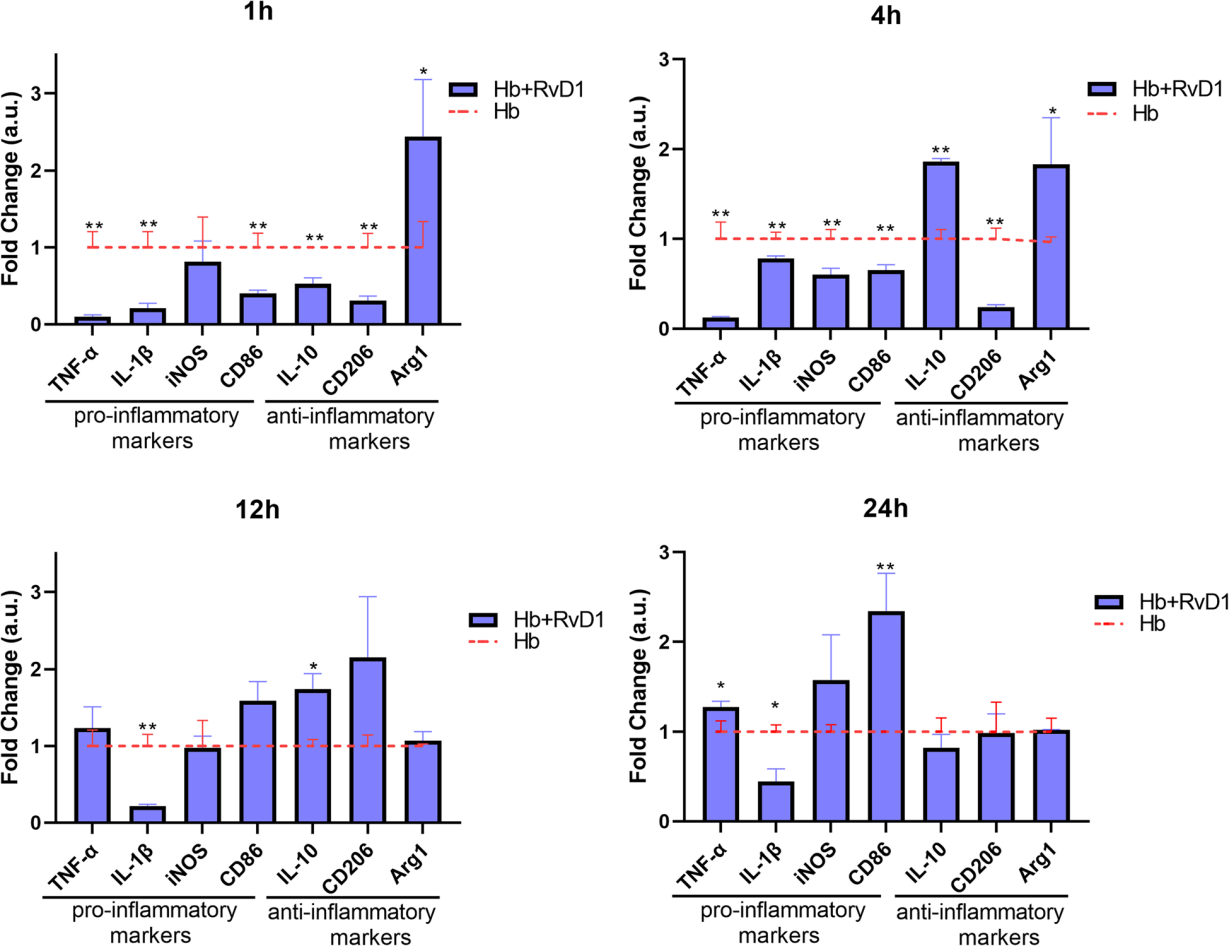
Effects of RvD1 on the polarization of Hb-stimulated microglia. Microglia were cultured in medium containing 25 nM RvD1 for 30 min, and then cultured in 20 μM Hb for 1–24 h (*n* = 3). The data showed the relative multiple changes in gene expression in the treatment group compared with that observed in the Hb group (baseline). Each gene at different time points was analyzed by Student’s *t* test and statistical significance determined using the Holm-Sidak method, with alpha = 0.05. Each gene at each time point was analyzed individually, without assuming a consistent SD. *p < 0.05 and **p < 0.01. *n* is the number of independent cell samples

After confirming that the SAH in vitro model was essentially consistent, we added 25 nM RvD1 to the culture medium for 30 min in advance to observe its effect on microglial cell polarization and performed qPCR to detect the mRNA expression changes. As shown in Fig. 3, for pro-inflammatory cytokines, RvD1 significantly inhibited the expression of TNF-α and IL-1β induced by Hb at 1 and 4 h. Especially for IL-1β, the inhibition effect lasted for 24 h, while TNF-α expression rebounded at 24 h. RvD1 also significantly inhibited the iNOS and CD86 expression. For iNOS, the inhibition was the most obvious at 4 h, with no subsequent significant difference observed at other time points. Regarding CD86 expression, significant attenuation was observed at 1 and 4 h, but at 24 h, a significant rebound was observed, similar to that detected for TNF-α. For IL-10, expression was significantly decreased by RvD1 at 1 h but increased significantly at 4 and 12 h, suggesting the potential effect of RvD1 to promote the transformation of the anti-inflammatory response. For CD206, RvD1 also significantly reduced its expression in the early period (1 and 4 h), but in the later period (12 and 24 h), no significant difference was observed compared with that detected in the Hb stimulation group. The expression of Arg1 was significantly promoted by RvD1 at 1 and 4 h, while no significant difference could be observed in the later phase. These results suggested that RvD1 indeed had a significant anti-inflammatory effect and that it could potentially promote the anti-inflammatory polarization of microglia.

### RvD1 inhibits the protein expression of TNF-α but promotes that of IL-10

**Fig. 4:**
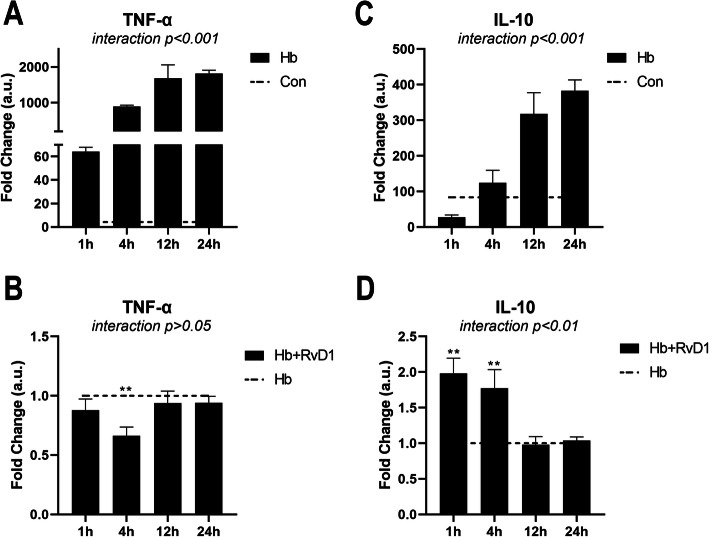
Protein expression pattern of TNF-α and IL-10 after the Hb and RvD1 treatment. **a**, **c** Time course of protein expression after Hb stimulation. Microglia were cultured in 20 μM Hb for 1 to 24 h (*n* = 3). **b**, **d** Effect of RvD1 on Hb-induced TNF-α and IL-10 expression at different time points. Microglia were cultured in medium containing 25 nM RvD1 for 30 min, and then cultured in 20 μM Hb for 1–24 h (*n* = 3). The data were analyzed by two-way ANOVA and Sidak post hoc multiple comparison. **p < 0.01. *n* is the number of independent cell samples

In the previous assays, we only assessed the polarization phenotype index, i.e., the changes in mRNA expression. As shown in Fig. 4, we also assessed the changes in the protein expression of TNF-α and IL-10 by ELISA. The protein expression of TNF-α and IL-10 was significantly promoted after Hb stimulation, which increased at 1 h and then gradually increased, peaking at 24 h (Fig. 4a, c). After RvD1 treatment, the protein expression of TNF-α was significantly inhibited at 4 h but not at other time points, while the protein expression of IL-10 significantly increased at 1 and 4 h, with no significant difference observed in the later phase (Fig. 4b, d). These results provided further evidence of the effects of RvD1 on microglia-related inflammation, suggesting that RvD1 could show a superior anti-inflammatory effect.

### RvD1 possibly functions by regulating the IRAK1/TRAF6/NF-κB signaling pathway

**Fig. 5:**
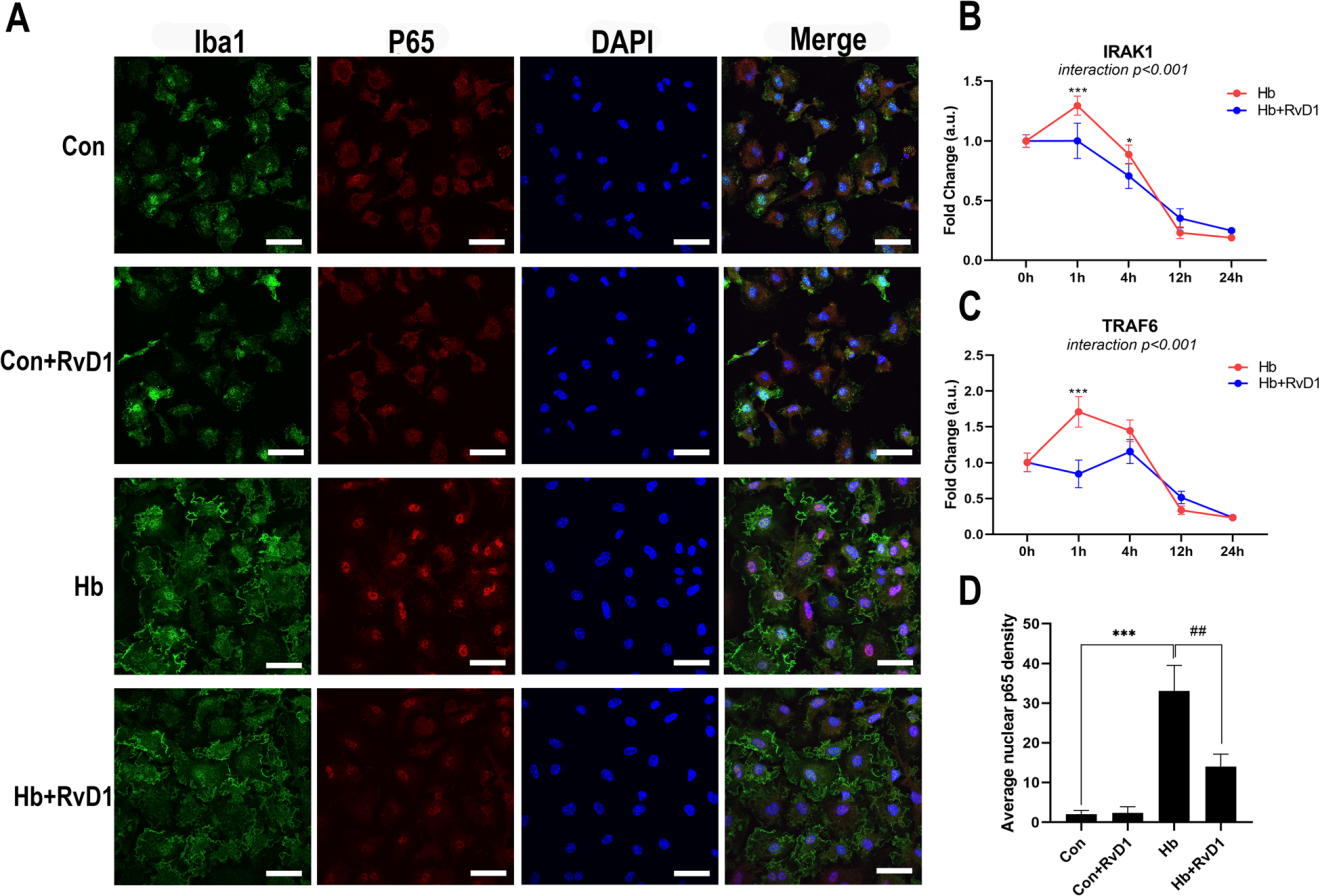
Effects of RvD1on the IRAK1/TRAF6/NF-κB signaling pathway. **a**, **d** The nuclear translocation of p65. Microglia were cultured in medium containing 25 nM RvD1 for 30 min, which was followed by 15 min of Hb stimulation (*n* = 3). **b**, **c** mRNA expression changes in IRAK1 and TRAF6 levels after RvD1 treatment. Microglia were cultured in medium containing 25 nM RvD1 for 30 min before being cultured in 20 μM Hb for 1–24 h (*n* = 3). Data were analyzed by one-way ANOVA followed by Tukey’s post hoc multiple comparison for **a**, **d** and two-way ANOVA followed by Sidak post hoc multiple comparison for **b**, **c**. *p < 0.05, ***p < 0.001, and ##*p* < 0.01. Bar = 40 μm. *n* is the number of independent cell samples

After confirming that RvD1 could have a good anti-inflammatory effect, we continued to assess its potential mechanisms. To this end, we used immunofluorescence staining and qPCR analyses to assess the changes in key proteins in the pro-inflammatory signaling pathways. Fifteen minutes after Hb stimulation, microglia showed obvious activation, as evidenced by the large amount of p65 nuclear translocation observed (Fig. 5a, d). However, after RvD1 treatment, p65 nuclear translocation was significantly inhibited. It was worth mentioning that although RvD1 inhibited the activation of this inflammatory pathway, the morphology of microglia was not significantly different from that of the Hb stimulation group, both of which showed an increase in mitosis and in cytoplasm volume appearing as “fried egg” (Fig. 5a). The mRNA expression was significantly inhibited at 1 h after RvD1 treatment for both IRAK1 and TRAF6, while only for IRAK1 at 4 h. Meanwhile, they both slightly rebounded at 12 h, with no significant difference observed (Fig. 5b, c).

### NF-κ B and MAPKs signaling activities are inhibited by RvD1

**Fig. 6:**
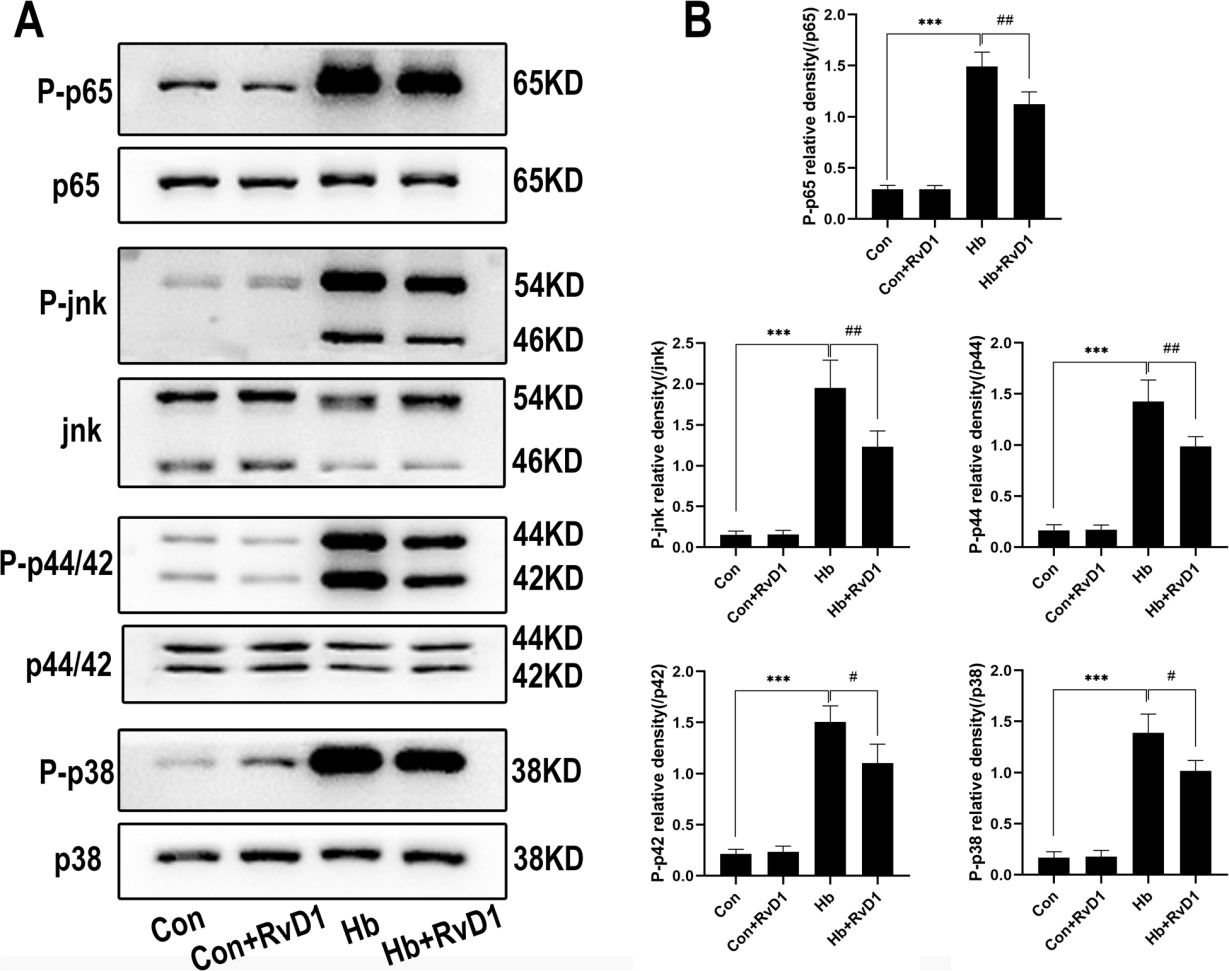
Effects of RvD1 on the protein expression in NF-κB or MAPKs signaling activities. Microglia were cultured in medium containing 25 nM RvD1 for 30 min, which was followed by 15 min of Hb stimulation (*n* = 3). **a** Western blot results for each protein. **b** The corresponding statistical results for **a**. The levels of p-p65, p-p38, p-JNK, p-p44, and p-p42 were expressed as the relative change to the corresponding total protein. The data were analyzed by one-way ANOVA and Tukey’s post hoc multiple comparison. ***p < 0.001, #*p* < 0.05, and ##*p* < 0.01. *n* is the number of independent cell samples

To further assess the inhibitory effects of RvD1 on the inflammatory pathways, we assessed the levels of proteins in the downstream of NF-κB and MAPKs pathways by western blot analysis. The results shown in Fig. 6 demonstrated that the proteins of the two pathways were obviously activated after Hb stimulation, suggesting that they could strongly promote the pro-inflammatory polarization of microglia. Although the phosphorylation levels of p65, JNK, p44, p42, and p38 remained higher than those of the control group after the application of RvD1 in the Hb + RvD1 group, the phosphorylation levels were significantly inhibited when compared with the Hb stimulation group, suggesting that RvD1 had a regulatory effect on both pathways. With respect to microglia, both pathways were regulated by IRAK1/TRAF6, which also confirmed the regulatory effects of RvD1 on upstream proteins.

### RvD1 ameliorates Hb-induced neuronal oxidative stress and synaptic damage

**Fig. 7:**
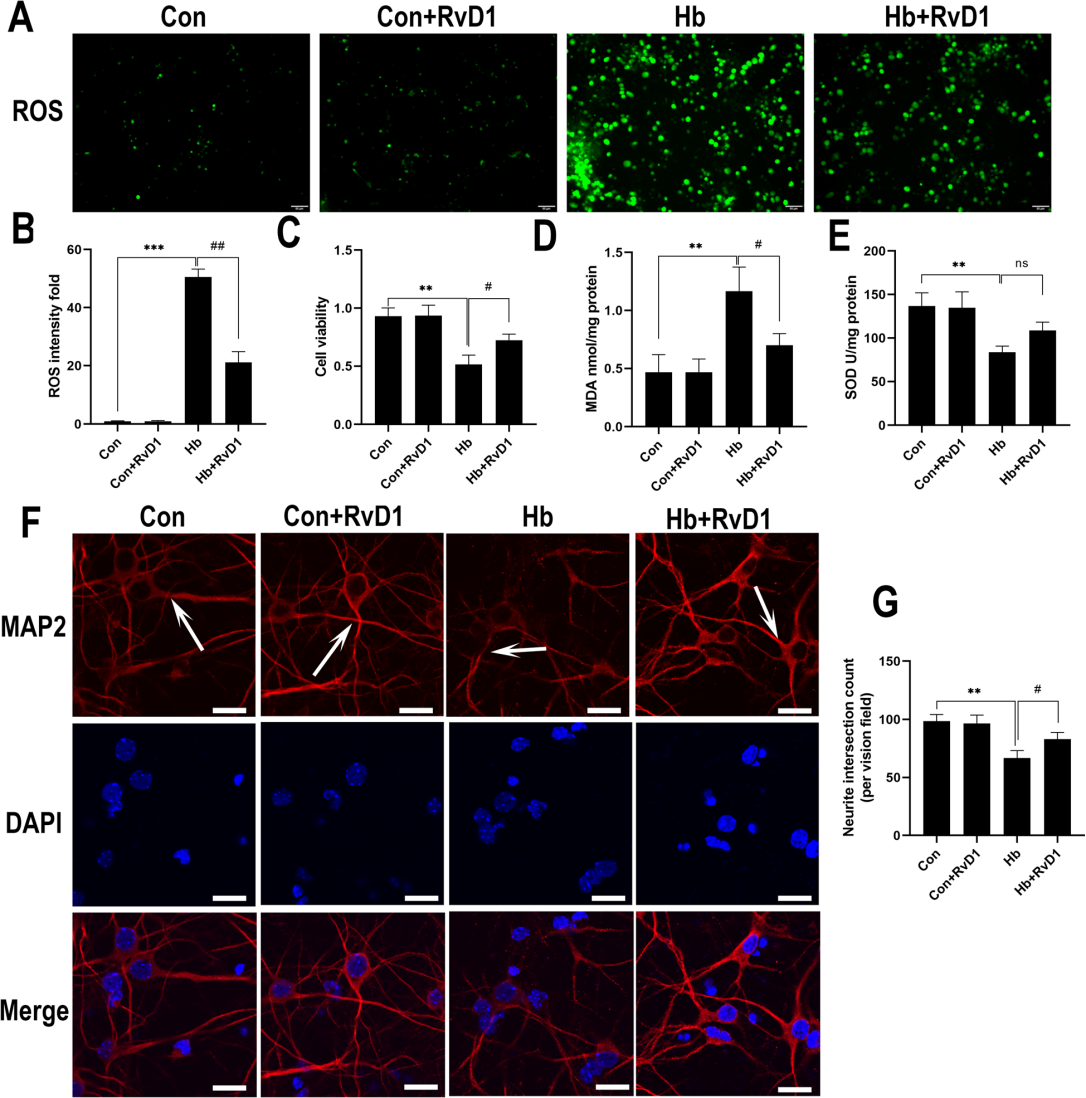
Effects of RvD1 on the oxidative stress and synaptic growth of neurons. The primary neurons were cultured in medium containing 50 μM Hb for 24 h, and RvD1 was added at a concentration of 25 nM 30 min before Hb stimulation. **a**, **b** The production of ROS in neurons and the statistical results for each group (*n* = 3). **c** The activity of neurons was measured by the CCK-8 method (*n* = 6). **d**, **e** MDA content and SOD activity (*n* = 3). **f** The synapse changes of neurons, shown as white arrows. **g** The number of synaptic intersections in the visual field analyzed by ImageJ (*n* = 5). The data were analyzed by one-way ANOVA and Tukey’s post hoc multiple comparison. **p < 0.01, ***p < 0.001, #*p* < 0.05, and ##*p* < 0.01. Bar = 50 μm for ROS staining and 40 μm for MAP2 staining. *n* is the number of independent cell samples

Oxidative stress of neurons is the primary factor associated with neuronal damage. As we observed that ALX/FPR2 was expressed in a large number of neurons, in this experiment, primary neurons were used to assess whether RvD1 had a direct effect on neurons. To this end, we observed the effects of RvD1 on the oxidative stress of neurons and synaptic damage after Hb treatment. ROS levels in neurons from the Hb-treated group were significantly higher than those of the control group, indicating that Hb caused notable oxidative stress, while ROS-positive cell staining significantly reduced after RvD1 application (Fig. 7a, b). The results of cell viability, MDA content, and SOD enzyme activity assays also showed similar results (Fig. 7c–e). After Hb treatment, the viability of primary neurons decreased, the MDA content increased, and SOD enzyme activity decreased. Compared with the Hb-treated group, the viability of cells significantly increased and the content of MDA decreased after the application of RvD1, and although SOD enzyme activity increased, no significant difference was observed. For the synaptic damage experimental results, we also observed that the synapses stained for microtubule-associated protein 2 (MAP2) significantly reduced after Hb treatment, while the application of RvD1 could reverse this damage (Fig. 7f, g).

### RvD1 can reduce the death of neurons in vitro caused by Hb

**Fig. 8:**
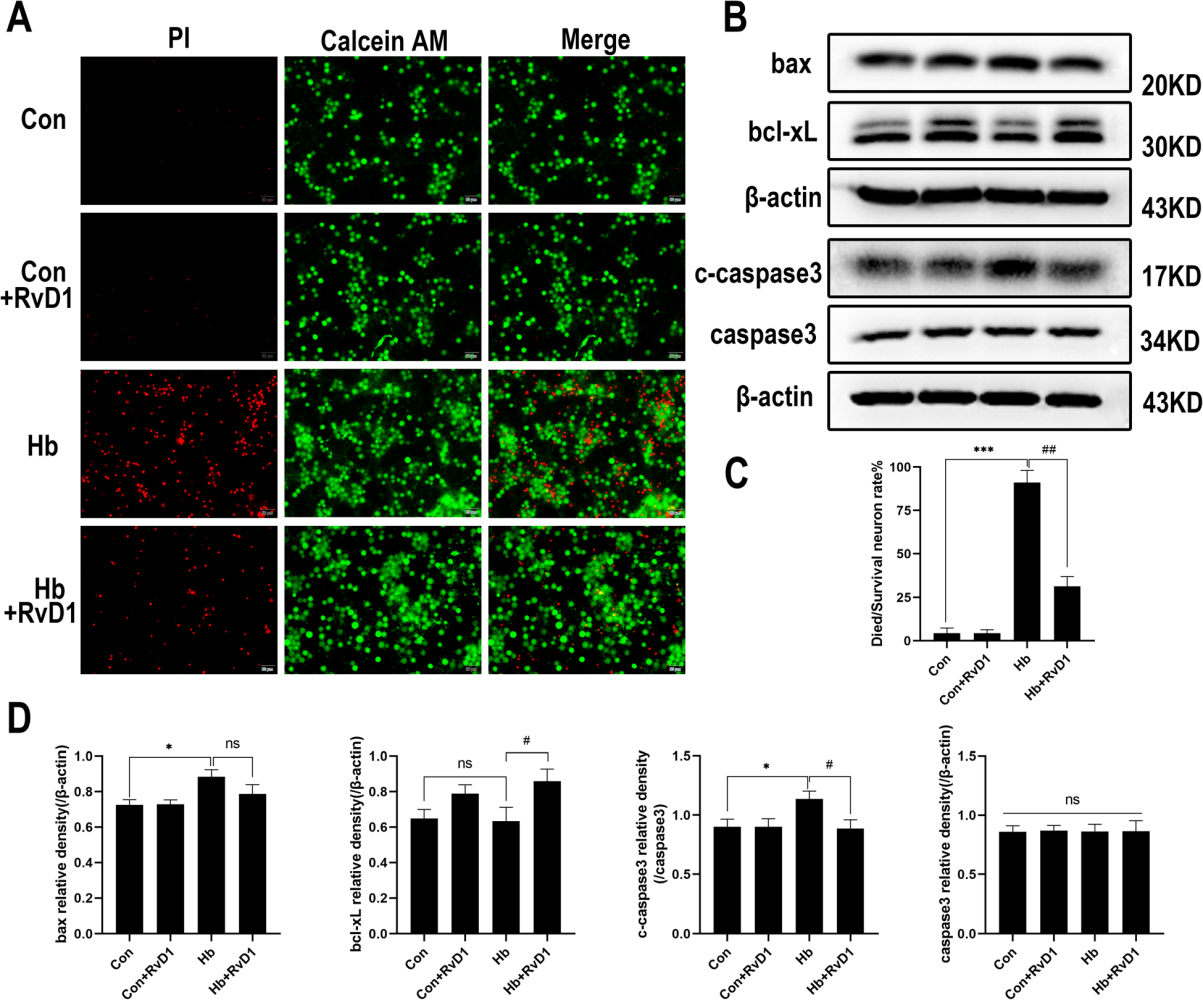
Influence of RvD1 on neuronal death induced by Hb. The primary neurons were cultured in a medium containing 50 μM Hb for 24 h, and RvD1 was added at a concentration of 25 nM 30 min before Hb stimulation. **a**, **c** Live-dead cell staining and the corresponding quantitative statistical results (*n* = 3). The red color shows PI staining, indicating dead cells; the green color shows calcein AM staining, indicating live cells. **b**, **d** The western blot results of Bax, bcl-xL, cleaved caspase-3, caspase-3, and the corresponding semi quantitative statistical results (*n* = 3). The data were analyzed by one-way ANOVA and Tukey’s post hoc multiple comparison. *p < 0.05, ***p < 0.001, #*p* < 0.05, ##*p* < 0.01 and ns showed no significant difference. n is the number of independent cell samples. Bar = 50 μm

The results showed that a large number of primary neurons died after Hb stimulation. As shown in Fig. 8a, c, numerous dead cells appeared in Hb-treated group, but significantly decreased after RvD1 application. The western blot results of apoptosis-related proteins showed that Hb stimulation caused an increase in bax and cleaved caspase-3 levels, but did not significantly downregulate bcl-xL and caspase-3. Compared with that observed in Hb stimulation group, the expression of bax protein decreased after the application of RvD1, but no significant difference was observed. Furthermore, the expression of bcl-xL protein significantly increased, while that of cleaved caspase-3 decreased, whereas total caspase-3 levels did not (Fig. 8b, d).

### RvD1-mediated effects on microglia and neurons are dependent on ALX/FPR2

**Fig. 9:**
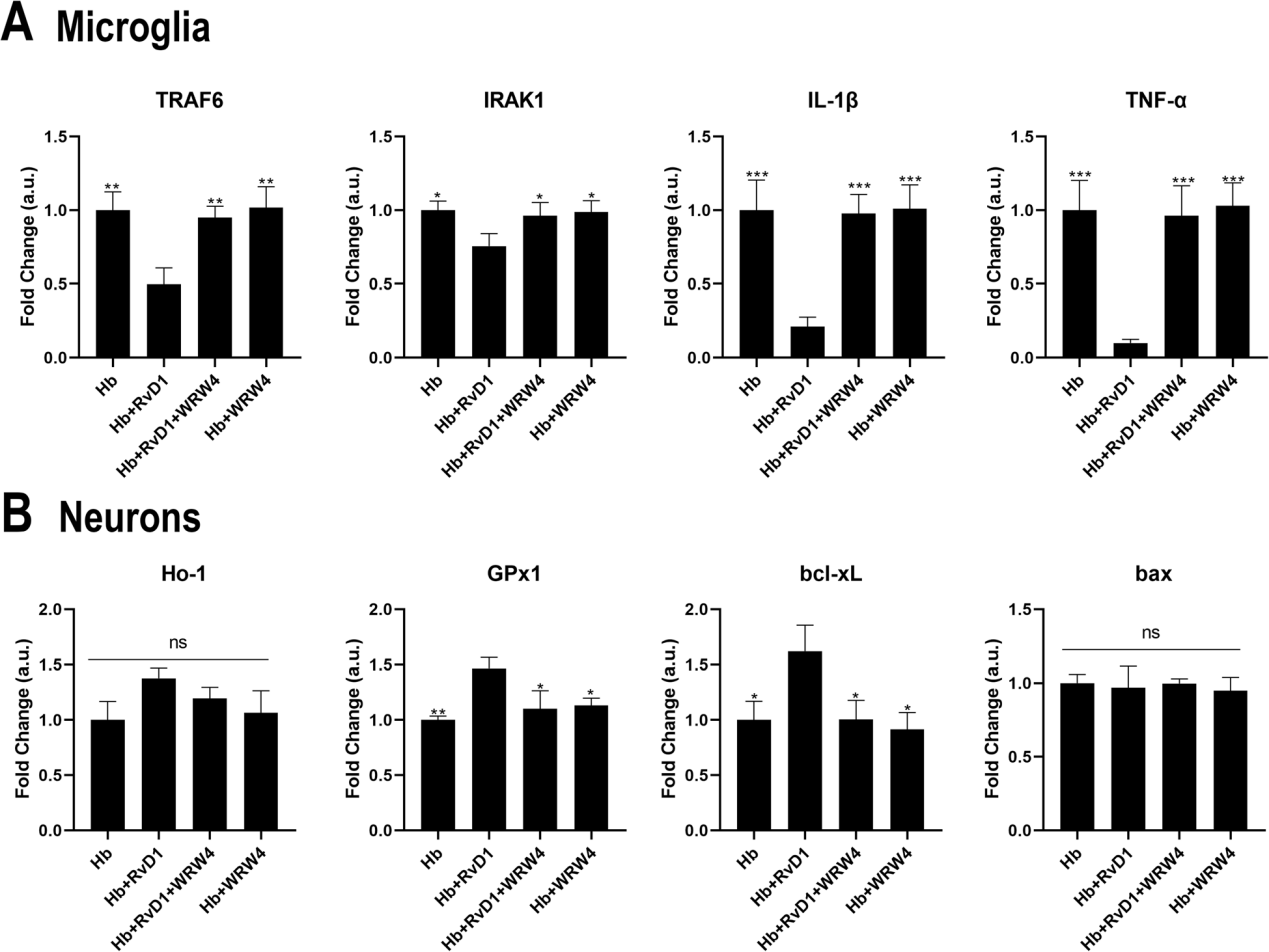
WRW4 reverses the effect of RvD1 on microglia and neurons. The ALX/FPR2 specific antagonist WRW4 (10 μM) and RvD1 (25 nM) were added 30 min before Hb stimulation, and microglia were then stimulated by 20 μM Hb for 1 h, while primary neurons were stimulated by 50 μM Hb for 12 h. **a** mRNA expression of IRAK1/TRAF6/IL-1β/TNF-α for microglia (*n* = 3). **b** mRNA expression of Ho-1/GPx1/bcl-xL/bax for neurons (*n* = 3). The data were analyzed by one-way ANOVA and Tukey’s post hoc multiple comparison. *p < 0.05, **p < 0.01, ***p < 0.001 compared with Hb + RvD1 group and ns showed no significant difference. *n* is the number of independent cell samples

We showed that microglia and neurons expressed the receptor ALX/FPR2 and observed some functions of RvD1 on primary microglia and neurons. To further confirm whether the effects of RvD1 are based on an RvD1-ALX/FPR2 interaction, we used the ALX/FPR2-specific antagonist WRW4 to investigate whether it can abolish the effects of RvD1. The results (Fig. 9a) showed that the mRNA expression of upstream signaling pathway genes (IRAK1/TRAF6) were reversed by addition of WRW4, as well as the downstream factors (IL-1β/TNF-α) when comparing the Hb + RvD1 group to the Hb + RvD1 + WRW4 group. With respect to neurons (Fig. 9b), the mRNA expression of the antioxidant gene glutathione peroxidase 1 (GPx1) and the anti-apoptosis gene bcl-xL were significantly abolished by WRW4. The expression of other genes such as heme oxygenase 1 (Ho-1) and bax showed no significant differences when comparing the various groups, indicating that they might not be good indicators of RvD1 activity.

## Discussion

In the present study, we investigated the expression pattern of ALX/FPR2 and observed the functions of the RvD1-ALX/FPR2 interaction in primary microglia and neurons. There are four primary findings of the present study: (1) ALX/FPR2 is largely expressed in neurons, with moderate expression observed in microglia and no expression in astrocytes; (2) RvD1 can inhibit the Hb-induced microglial pro-inflammatory response and possibly promote anti-inflammatory polarization; (3) the anti-inflammatory effects of the RvD1-ALX/FPR2 interaction potentially occurs through regulation of IRAK1/TRAF6 signaling activities; (4) RvD1 has the potential to inhibit Hb-induced neuronal damage or apoptosis.

ALX/FPR2 has been widely studied on neutrophils and monocytes. It is a promiscuous receptor that can bind to many types of ligands and exert different functions [15]. In the CNS, Aβ protein phagocytosis via ALX/FPR2 was investigated in the Alzheimer’s disease model [43]. However, the cell localization of ALX/FPR2 has seldom been explored in the brain. The results of our in vivo and in vitro experiments showed that ALX/FPR2 was highly expressed in neurons, moderately expressed in microglia, and not expressed in astrocytes. The ALX/FPR2 expression results in neurons were consistent with the findings of a study by Ho [31, 32], providing a theoretical basis and research direction for studying the analgesic effects of RvD1 [44, 45]. For microglia, the observed ALX/FPR2 expression was essentially the same as that observed in a number of other studies [46–48], but the lack of ALX/FPR2 expression in astrocytes was different from the results of other studies [49, 50]. However, these tissue immunofluorescence results were not fully observed in the present study, which might be due to the high expression of neurons covering up the expression of microglia.

ALX/FPR2 expression increased significantly after SAH and maintained from 24 h to approximately 3 days, suggesting that ALX/FPR2 might play an important role in the pathophysiological process after SAH. Based on the published studies of ALX/FPR2 [43, 46, 48] and our above results, it might also be feasible to use the ALX/FPR2 as a potential target in the treatment of SAH.

The polarization phenotype of microglia induced by Hb indicated that it was not totally opposite about the polarization of pro-inflammatory and anti-inflammatory phenotypes. Indeed, it has been shown that NF-κB plays dual roles in the acute and resolution stages of inflammation, because it not only increases the levels of pro-inflammatory factors but also promotes the expression of anti-inflammatory factors [51]. In the present study, the same results were also observed. Specifically, in the early phase (1 and 4 h), when the expression of TNF-α and IL-1β significantly increased, the expression of IL-10 and CD206 also significantly increased. These results suggested that the polarization of microglia was complex even under the stimulation of a single factor, and that the polarization direction continued to change with time. Nevertheless, a weakness of the present study is that the indicators could not fully assess the polarization phenotype, indicating that more samples and indicators are needed to verify these results in future.

After the application of RvD1, the polarized phenotype of microglia further changed. The expression of pro-inflammatory markers was significantly inhibited in the early stage, where IL-1β was inhibited throughout the time course, while some pro-inflammatory markers such as TNF-α, iNOS and CD86 rebounded to different degrees over 24 h. These results confirmed those of earlier studies performed by Serhan et al. and Hong et al. showing that RvD1 acts on microglia to reduce cytokine production [5, 52]. For the anti-inflammatory index, IL-10 and CD206 were also significantly inhibited after 1 or 4 h but increased in the later period. Among them, IL-10 was significantly different between the Hb treatment and RvD1 groups, while the levels of CD206 were not significantly different. Arg1 was markedly upregulated in the early stage and gradually downregulated in the later stage. These results further confirmed the complexity of microglial polarization. However, it was undeniable that RvD1 had a significant regulatory effect on microglia, especially in the inhibition of pro-inflammatory phenotype. When we further assessed the protein expression changes of TNF-α and IL-10, increased IL-10 levels were observed at 1 and 4 h, which was essentially consistent with the previous results. In contrast, TNF-α was significantly inhibited at 4 h, although it could also explain the inhibitory effects of RvD1, but the effective time course was too short, suggesting that post transcriptional regulation or protein level modification were involved.

Subsequently, we continued to assess the expression or activation of upstream and downstream proteins of pro-inflammatory factors to elucidate the underlying mechanisms. The results showed that the transcriptional expression of TRAF6 and its upstream factor IRAK1 was upregulated by Hb and the Hb-induced upregulation was inhibited by RvD1. The activation of many proteins in the downstream NF-κB and MAPKs pathways also showed similar changes, which was essentially consistent with the observed phenotypic change. All of the above data indicated that RvD1 could inhibit the microglial pro-inflammatory response, possibly by regulating IRAK1/TRAF6 signaling activities.

Neuronal death is an important pathological phenomenon after SAH and is also the most studied aspect of this process. In the present study, we observed the effects of RvD1 on neuronal apoptosis and oxidative stress after SAH in vitro. We confirmed that RvD1 had a direct protective effect on primary neurons, which was primary reflected in the improvements in cell viability, inhibition of the production of oxidative stress products, the avoidance of synaptic injury and a reduction in cell death induced by Hb. These results were consistent with those of previous studies [50, 53, 54]. For example, Peritore et al. observed that ALX/FPR2 gene knockout significantly increased the apoptosis of brain neurons in a depression model [50]. The results of some studies also suggested other potential factors. For instance, He et al. showed that ALX/FPR2 could mediate neuronal apoptosis, which was inhibited by WRW4, a specific inhibitor of ALX/FPR2 [55]. In addition, Ying et al. observed that ALX/FPR2 was expressed in neuronal cell lines, which could increase the susceptibility of these cells to Aβ protein, while other ALX/FPR2 ligands, such as humanin and W peptide, could inhibit this susceptibility and reduce Aβ-induced neuronal apoptosis [53]. Based on the results of the present study and other studies, we can draw a preliminary conclusion that ALX/FPR2 is expressed in neurons, but its specific function is determined by ligands, where ALX/FPR2 can promote either neuronal apoptosis or neuron growth through binding different ligands.

It was not investigated how ALX/FPR2 mediated the protective effects of RvD1 on neurons in the present study. RvD1 could promote bcl-xL expression, meanwhile, the inhibitory effects of RvD1 were also observed on the increase in cleaved caspase-3 and bax levels induced by Hb. Regarding the related proteins upstream of the ALX/FPR2 signaling pathway, no detailed assessments were made, which was also a deficiency of the present study and would be further addressed in future studies. However, the results of some other relevant studies might provide some indications. For instance, Fan et al. showed that RvD1 functioned through the PI3K-Akt-caspase-3 pathway in rats with cerebral hemorrhage in vivo and neuronal Hb stimulation in vitro [54]. However, there are few relevant results for other signaling pathways, which need further study in future.

Some additional limitations of the present study include the small sample size and a follow-up study with a large sample size is necessary. In addition, animal experiments are needed to further explore the role of the RvD1-ALX/FPR2 interaction in the CNS.

## Conclusion

**Fig. 10:**
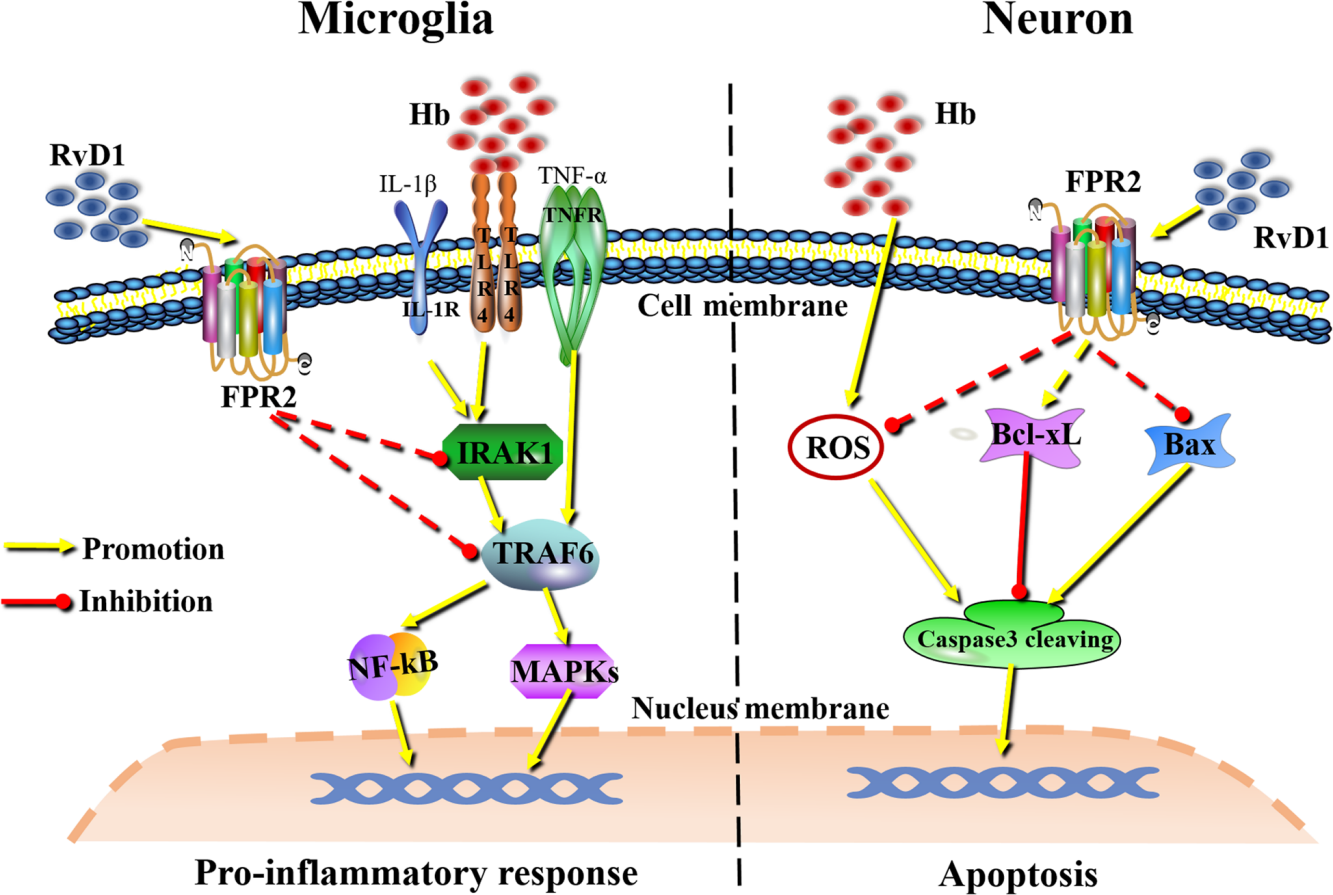
Graphical summarization of the functions of the RvD1-ALX/FPR2 interaction on microglia and neurons. The RvD1-ALX/FPR2 interaction can inhibit microglial pro-inflammatory polarization by negatively regulating IRAK1/TRAF6 signaling activities. The RvD1-ALX/FPR2 interaction can also ameliorate Hb-induced neuronal oxidative stress and death in neurons

In the present study, ALX/FPR2 was shown to be expressed in neurons and microglia. The RvD1-ALX/FPR2 interaction exerted superior inhibitory effects on Hb-induced microglial pro-inflammatory polarization, possibly by negatively regulating the signaling activities of IRAK1/TRAF6/NF-κB or MAPKs. With respect to neurons, the RvD1-ALX/FPR2 interaction could also ameliorate Hb-induced neuronal oxidative damage and death (Fig. 10). All of the above results indicated a novel therapeutic target (ALX/FPR2) and drug (RvD1) for the treatment of SAH and other associated diseases.

## Supplementary information

**Additional file 1: Figure S1.** Chemical structure of RvD1. **Figure S2.** Dose response experiments of RvD1 in microglia and neurons. The primary microglia and neurons were cultured in a medium containing 20μM or 50μM Hb for 12 hours, respectively. RvD1 was added at a concentration of 25nM or 75nM 30 minutes before Hb stimulation. A TNF-α mRNA expression changes in microglia. B bcl-xL mRNA expression changes in neurons. The data were analyzed by one-way ANOVA and Tukey’s post hoc multiple comparison. **p<0.01, #*p*<0.05 and ns showed no significant difference. n is the number of independent cell samples.

## Data Availability

The datasets supporting the conclusions of this article are included within the article.

## Abbreviations

SAH: Subarachnoid hemorrhage
EBI: Early brain injury
RvD1: Resolvin d1
ALX/FPR2: Lipoxin A4 receptor/formyl peptide 2
CNS: Central nervous system
WRW4: Trp-Arg-Trp-Trp-Trp-Trp-NH2
Hb: Hemoglobin
IL-1β: Interleukin 1 beta
TNF-α: Tumor necrosis factor alpha
IL-10: Interleukin 10
iNOS: Induced nitric oxide synthases
GAPDH: Glyceraldehyde-3-phosphate dehydrogenase
CD86: Cluster of Differentiation 86
Arg1: Arginase 1
CD206: Cluster of differentiation 206
TRAF6: TNF receptor-associated factor 6
IRAK1: Interleukin-1 receptor-associated kinase 1
NF-κB: Nuclear factor-ĸB
MAPKs: Mitogen-activated protein kinases
LPS: Lipopolysaccharide
IF: Immunofluorescence
ROS: Reactive oxygen species
bcl-xL: Bcl2 like 1
Bax: Bcl2-associated x
Gpx1: Glutathione peroxidase 1
Ho-1: Heme oxygenase 1
RRIDs: Research Resource Identifiers
