# Supplementary material for: Functions of resolvin D1-ALX/FPR2 receptor interaction in the hemoglobin-induced microglial inflammatory response and neuronal injury
Source: J Neuroinflammation. 2020 Aug 14;17:239. doi: 10.1186/s12974-020-01918-x (PMC7429751; doi:10.1186/s12974-020-01918-x)

Figure S1 Chemical structure of RvD1.

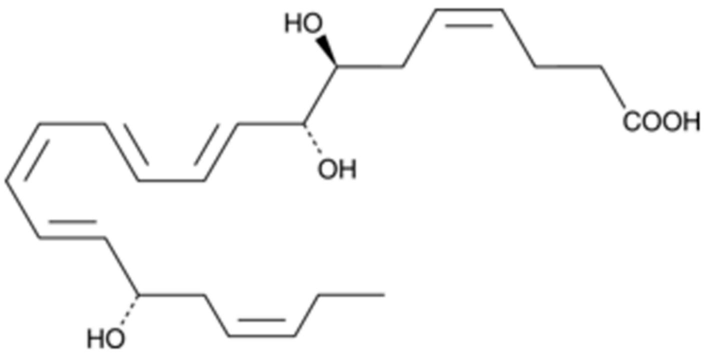

Figure S2 Dose response experiments of RvD1 in microglia and neurons.

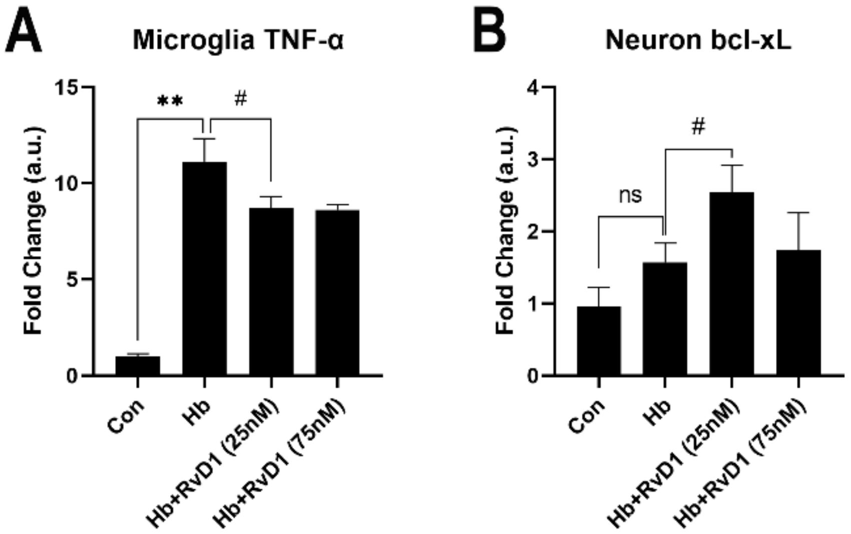

Supplement: Supplementary file 1 — Additional file 1: Figure S1. Chemical structure of RvD1. Figure S2. Dose response experiments of RvD1 in microglia and neurons. The primary microglia and neurons were cultured in a medium containing 20μM or 50μM Hb for 12 hours, respectively. RvD1 was added at a concentration of 25nM or 75nM 30 minutes before Hb stimulation. A TNF-α mRNA expression changes in microglia. B bcl-xL mRNA expression changes in neurons. The data were analyzed by one-way ANOVA and Tukey’s post hoc multiple comparison. **p<0.01, #p<0.05 and ns showed no significant difference. n is the number of independent cell samples. [file 12974_2020_1918_MOESM1_ESM.pdf]
